# Regulation of retinal membrane guanylyl cyclase (RetGC) by negative calcium feedback and RD3 protein

**DOI:** 10.1007/s00424-021-02523-4

**Published:** 2021-02-03

**Authors:** Alexander M. Dizhoor, Igor V. Peshenko

**Affiliations:** Pennsylvania College of Optometry, Salus University, 8360 Old York Road, Elkins Park, PA 19027 USA

**Keywords:** Guanylyl cyclase (Guanylate cyclase), Photoreceptors, Retinal degeneration, GUCY2D, GCAP, RD3

## Abstract

This article presents a brief overview of the main biochemical and cellular processes involved in regulation of cyclic GMP production in photoreceptors. The main focus is on how the fluctuations of free calcium concentrations in photoreceptors between light and dark regulate the activity of retinal membrane guanylyl cyclase (RetGC) via calcium sensor proteins. The emphasis of the review is on the structure of RetGC and guanylyl cyclase activating proteins (GCAPs) in relation to their functional role in photoreceptors and congenital diseases of photoreceptors. In addition to that, the structure and function of retinal degeneration-3 protein (RD3), which regulates RetGC in a calcium-independent manner, is discussed in detail in connections with its role in photoreceptor biology and inherited retinal blindness.

## Introduction

**Fig. 1:**
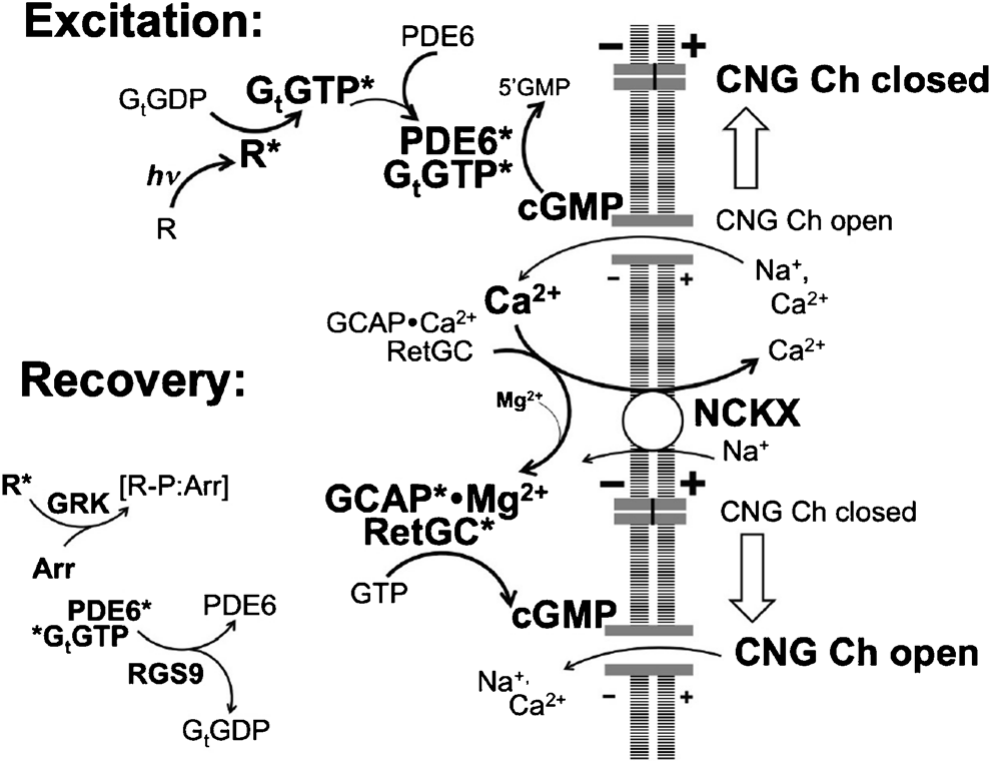
The schematic of Ca^2+^ feedback and guanylyl cyclase regulation in photoreceptor. During excitation phase, photoactivated pigment (R*) activates the transduction cascade, R*➔Gt*➔PDE6*, which closes CNG channels in the outer segments and hyperpolarizes the photoreceptor membrane. Once the channels are closed, Ca^2+^ concentrations fall and thus allow Mg^2+^ to replace Ca^2+^ in GCAP. The Mg^2+^ GCAP stimulates cGMP production while the R* is being phosphorylated and blocked by arrestin and the transduction cascade is quenched by accelerated GTP hydrolysis in Gt, all resulting in reopening of the channels and restoring the inward cation current partially depolarizing photoreceptor; asterisks symbolize the activated state. *Arr* arrestin, *GCAP* guanylyl cyclase activating protein, *Gt* G protein transducin, *CNG Ch* cyclic nucleotide-gated channel, *GRK* G-protein receptor kinase, *NCKX* Na^+^/Ca^2+^, K^+^-exchanger, *PDE6* cGMP phosphodiesterase 6, *RGS9* regulator of G-protein signaling 9-1, *RetGC* retinal membrane guanylyl cyclase. See text for other details

Synthesis of cGMP is an essential part of the fundamental phototransduction process that defines the function of vertebrate rods and cones. Many excellent review articles describing the details of the phototransduction pathway have been published, to name a few, references [8, 9, 35, 52, 100, 106]. For that reason, we provide here only a brief overview of the signaling events that mediate absorption of a photon by visual pigment and the hyperpolarization of photoreceptor, the origin of the visual signaling (Fig. 1). Cyclic GMP plays a central role in phototransduction by regulating the permeability of the cyclic nucleotide-gated channels (CNG) in the plasma membrane of the photoreceptor’s outer segment. In the dark, retinal membrane guanylyl cyclase (RetGC) [25, 66, 125] produces enough cGMP to keep a small fraction of CNG channels in the open state, and that allows the channels to maintain an inward current of Na^+^ and Ca^2+^, partially depolarizing the photoreceptor membrane. Light activates cGMP hydrolysis by cGMP phosphodiesterase-6 (PDE6) through GDP/GTP exchange in the Gt protein, transducin, catalyzed by photoactivated rhodopsin or cone pigments. As GTP transducin activates PDE6, the rapid decline in cGMP concentration closes the CNG channels and stops the influx of Na^+^ and Ca^2+^ in the outer segment, thus causing hyperpolarization of rods and cones. The recovery of photoreceptors from excitation includes multiple steps, regulating all components of the phototransduction cascade. G-protein-coupled receptor kinases GRK1 and GRK7 phosphorylate photoactivated pigments to allow them to bind arrestins, which blocks activation of Gt; the intrinsic Gt GTPase activity accelerated by RGS9 protein and PDE6 rapidly deactivates Gt and thus returns its effector enzyme PDE6 to its inactive self-inhibited state, which halts cGMP decay. Quenching the rhodopsin-transducin-PDE6 cascade is a necessary, but not the only, step in photoreceptor recovery: timely re-opening of the CNG channels also requires acceleration of cGMP synthesis by RetGC via a negative Ca^2+^ feedback mechanism [52, 101].

The main purpose of this short review is to discuss the molecular mechanisms of the Ca^2+^ feedback with an emphasis on its role in regulation of cGMP synthesis as a part of normal photoreceptor physiology and pathological processes underlying congenital blindness. In addition to that, we will review a recently emerged role of a non-Ca^2+^ sensor protein, retinal degeneration 3 (RD3), as a potent regulator of RetGC function in photoreceptors and its role in protecting rods and cones from degeneration. The authors do not intend to provide a comprehensive detailed review of Ca^2+^ and guanylyl cyclase regulation in photoreceptors, but choose instead to present their opinion on only some key aspects of such regulation in the physiology and disease of photoreceptors.

## The origin of Ca^2+^ feedback in photoreceptors

Ca^2+^ feedback originates from fluctuations of intracellular Ca^2+^ in the outer segment between light and dark. Although the levels of free Ca^2+^ concentrations reported in the literature slightly vary between different measurements and different animal species, they all indicate a steep, up to tenfold, decline of free Ca^2+^ in response to light. Ca^2+^ continuously extruded from the outer segment via Na^+^/Ca^2+^, K^+^ exchangers, NCKX1 in rods, and NCKX2/NCKX4 in cones [116, 117], returns to the outer segment through the open CNG channels (Fig. 1), which keeps the free Ca^2+^ concentrations in dark-adapted photoreceptors in the range of ~250–560 nM [37, 121]. When the CNG channels close in light, the influx of Ca^2+^ into the outer segment stops and its free concentrations rapidly decline to the range of ~20–50 nM [37, 121]. Consequently, a number of processes regulated by Ca^2+^-binding proteins become affected, thus contributing to the shaping of photoresponses in rods and cones through negative Ca^2+^ feedback [35, 52, 100, 101]. Regulation of retinal membrane guanylyl (guanylate) cyclase (RetGC) by Ca^2+^ sensor proteins plays the major role in Ca^2+^ feedback [101].

## Ca^2+^ sensors in photoreceptors are recoverin-like EF-hand proteins

**Fig. 2:**
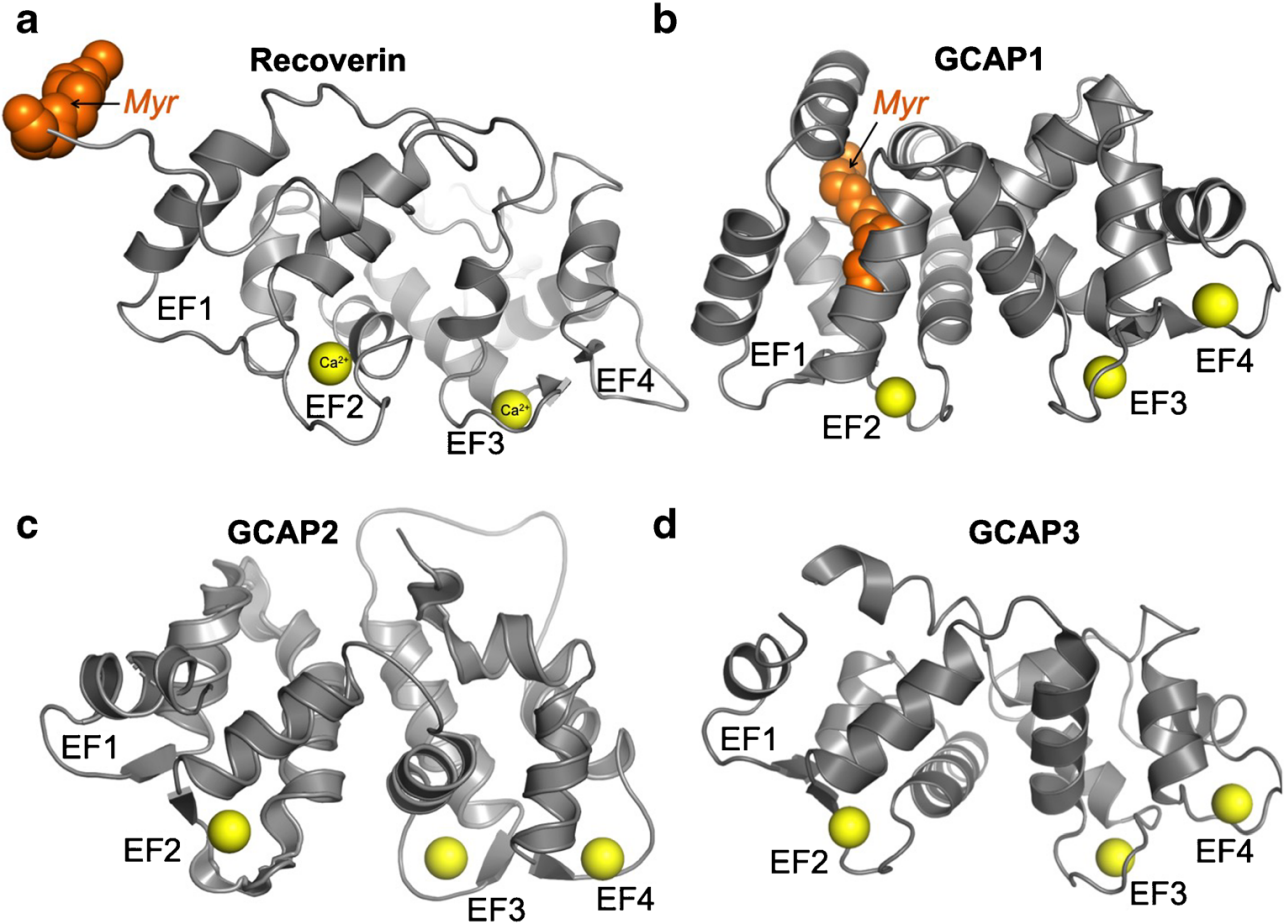
Ribbon diagrams of four Ca^2+^-liganded sensor proteins of the recoverin family. **a** Myristoylated recoverin [3, 33], **b** myristoylated GCAP1 [111], **c** non-myristoylated GCAP2 [6], and **d** non-myristoylated GCAP3 [110]. EF1 through EF4–EF-hands; *Myr* fatty acyl moiety; yellow spheres indicate the positions of metal ions in the EF-hand loops. Hereafter, the images were created using Shrödinger PyMol software by OpenGL version 2.0

**Fig. 3:**
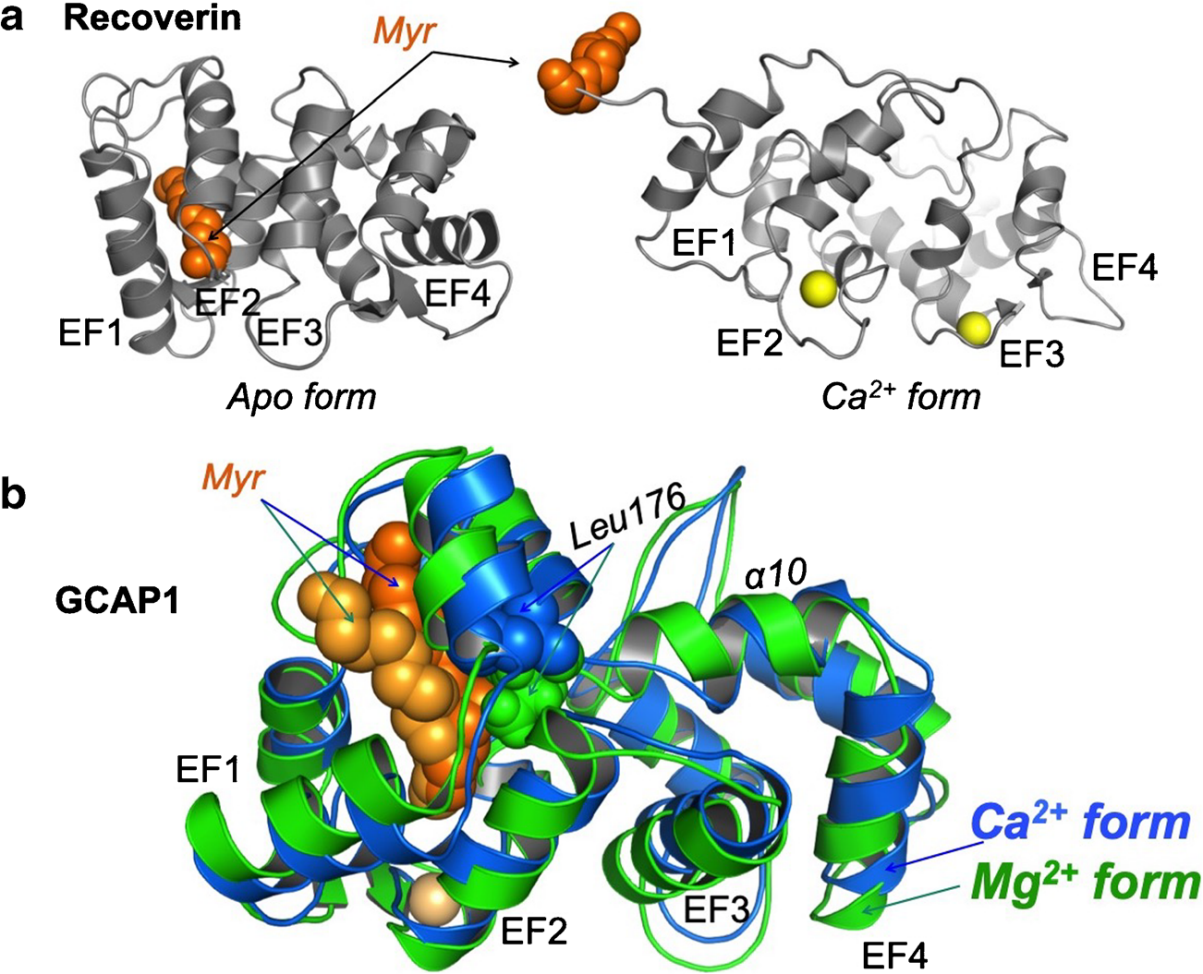
Ca^2+^-dependent conformational changes in recoverin and GCAP1. **a** Calcium-myristoyl switch in recoverin. In the apo form, the fatty acyl residue is buried inside the semiglobule comprised of EF1 and EF2. Upon transition from the apo form to the Ca^2+^-liganded state, the fatty acylated N-terminus becomes expelled from the protein structure [3]. **b** Calcium-myristoyl tug [91] in GCAP1, NMR structures of Mg^2+^ (green)—and Ca^2+^ (blue)—liganded states; NMR model by Lim et al. [63]. The myristoyl fatty chain remains constrained in the fold of the N-terminal lobe of the protein in the both physiological metal-liganded states, but changes its orientation as a result of the push-pull action of the exiting helix of EF-hand 4 transmitted via Leu-176 by partial unwinding/rewinding of the alpha-helix 10 [61, 63, 91]. See detailed explanations in the text

Proteins that mediate Ca^2+^ feedback belong to a distinct group of recoverin-like proteins in the EF-hand protein superfamily [13, 14]. The recoverin-like proteins harbor some notable features separating them from other groups of the EF-hand superfamily. To date, several photoreceptor-specific Ca^2+^-sensor proteins have been identified: recoverin (or S-modulin in lower vertebrate species), visinin, and guanylyl cyclase activating proteins (GCAPs) [13, 14, 57]. The latter subgroup includes several homologs, GCAP1 through GCAP8, in different vertebrate species [26, 43, 44, 81]. However, only two—GCAP1 and GCAP2—are ubiquitous among the animal species, and in some species, they are the only two GCAP isoforms present in photoreceptors [126]. A subpopulation of S-cones in humans also expresses GCAP3 [43]. Another EF-hand protein, GCIP [59], has been suggested to also play a physiological role in Ca^2+^ feedback, but later found not to be involved in shaping photoresponses [15, 72]. The recoverin-like Ca^2+^ sensor proteins have a number of common features: they have similar size (~21–25 kDa), are fatty acylated at the N-terminus, and have four helix-loop-helix EF-hand motifs, from EF-hand 1 (proximal to the N-terminus in primary structure) to EF-hand 4 (proximal to the C-terminus) [13, 14, 57] (Figs. 2, 3). The characteristic shape of the recoverin-like protein, distinct from other families of EF-hand proteins, presents two semi-globular lobes, each formed by a pair of EF-hand structures, connected via a “hinge” Gly residue [13, 57, 62]. In contrast to calmodulin-like proteins, the recoverin-like proteins do not undergo a major rearrangement of their core structures in response to binding and release of Ca^2+^ [62] (Figs. 2, 3).

The N-fatty acyl residue plays different structural roles in the function of photoreceptor Ca^2+^-sensor proteins. The N-fatty acyl groups in recoverin and GCAPs expressed in photoreceptors include saturated and non-saturated C12 and C14 residues, among which the saturated C14:0 myristoyl chain represents a relatively minor component [23, 81]. Despite that, the N-fatty acylation is commonly referred to as “myristoylation” for simplicity. In recoverin, the myristoyl group changes its position in protein structure: in a metal-free state of recoverin, the myristoyl residue is held inside the N-terminal lobe of the protein, but swings out when EF-hand 2 binds Ca^2+^ [4, 5, 24, 33, 128] (Fig. 3a). This change, commonly referred to as “calcium-myristoyl switch” [128, 130], has been characterized by detailed structural studies [5, 33]. The extrusion of the fatty acyl moiety increases recoverin’s affinity for the membrane [24], evidently by utilizing the extruded fatty chain as an anchor. Although the structures of visinin and S-modulin were not studied as comprehensively as that of recoverin, their primary structures are very closely homologous to that of recoverin [48], so both proteins are likely to share common three-dimensional characteristics with recoverin.

In contrast to recoverin, the myristoyl fatty chain in GCAPs remains buried inside the N-terminal semi-globule of the protein (Figs. 2, 3), regardless of its cation-liganded state [39, 60, 77, 111]. Instead of engaging in a calcium-myristoyl switch, the structural role of the myristoyl residue in GCAP1 is to link the N-terminal portion of the protein with its C-terminal lobe internally, via a structural link called the “calcium-myristoyl tug” (Fig. 3b) that plays an important role in fine-tuning GCAPs’ affinity for both Ca^2+^ and the target enzyme [91] as will be reviewed later in more detail.

Another common feature shared by the photoreceptor Ca^2+^ sensor proteins is that not all four EF-hand motifs in their primary structure are the actual metal binding domains [62]. The N-terminal EF-hand 1 in all proteins of the recoverin family lacks some of the essential oxygen-containing side chains required for coordination of Ca^2+^ [13, 14, 62] (Fig. 2). Recoverin has only two functional Ca^2+^-binding EF-hands (EF-hand 2 and-3), whereas three EF-hands (-2, -3 and -4) in GCAPs bind Ca^2+^ or Mg^2+^ [83] and play a major role in the GCAP-dependent regulation of the photoreceptor guanylyl cyclase, as described in the following sections.

## Metal binding properties of the photoreceptor-specific EF-hand sensor proteins

A possible function of recoverin is to help rhodopsin, by extending the life of its active state, to more effectively propagate activation of the phototransduction cascade in dim light, before the receptor is quenched by phosphorylation and arrestin binding [1, 7, 16, 47, 48]. GRK1 is considered to be the primary target for Ca^2+^ recoverin [1, 129] although a more direct effect on PDE6 activity was also recently proposed [75] (Fig. 1). In dark-adapted photoreceptors, recoverin can more effectively stick to the disk membranes as a result of its anchoring via calcium-myristoyl switch [17, 24, 129, 130]. Recoverin could also be the intracellular Ca^2+^ buffer in photoreceptors, although its contribution to the overall Ca^2+^-buffering capacity of photoreceptors has been estimated to be relatively modest [67].

Although the details of the functional role of GCAPs in regulation of the photoresponse require further study, it has been generally well established. GCAPs control the activity of RetGC in a Ca^2+^-sensitive manner by activating RetGC in the light, when Ca^2+^ concentrations fall, and decelerating the cyclase in the dark, when Ca^2+^ influx through the open CNG channels elevates free Ca^2+^ levels in the outer segment [35, 52, 100, 101, 106] (Fig. 1). GCAP-dependent regulation of the cyclase dominates the Ca^2+^ feedback, contributing to the feedback much stronger than other regulatory components of the photoresponse affected by Ca^2+^ [101]. Therefore, the metal-binding properties of GCAPs are especially important for their role in photoreceptors. GCAPs regulate RetGC within the physiological submicromolar range of free Ca^2+^ [82]. The affinities of EF-hands for Ca^2+^ in GCAPs are also very close to the physiological levels of free Ca^2+^ in the outer segment [83, 84]. However, the reported estimated *K*_d_ values of the EF-hands for Ca^2+^ vary rather widely depending on the methods used for their evaluation. Below, we examine in more detail how the metal binding properties of individual EF-hands in GCAP1 relate to the sensor function of GCAPs as the RetGC regulators. We will also address possible reasons for the variability between different estimated EF-hand affinities for Ca^2+^ in GCAP1.

**Fig. 4:**
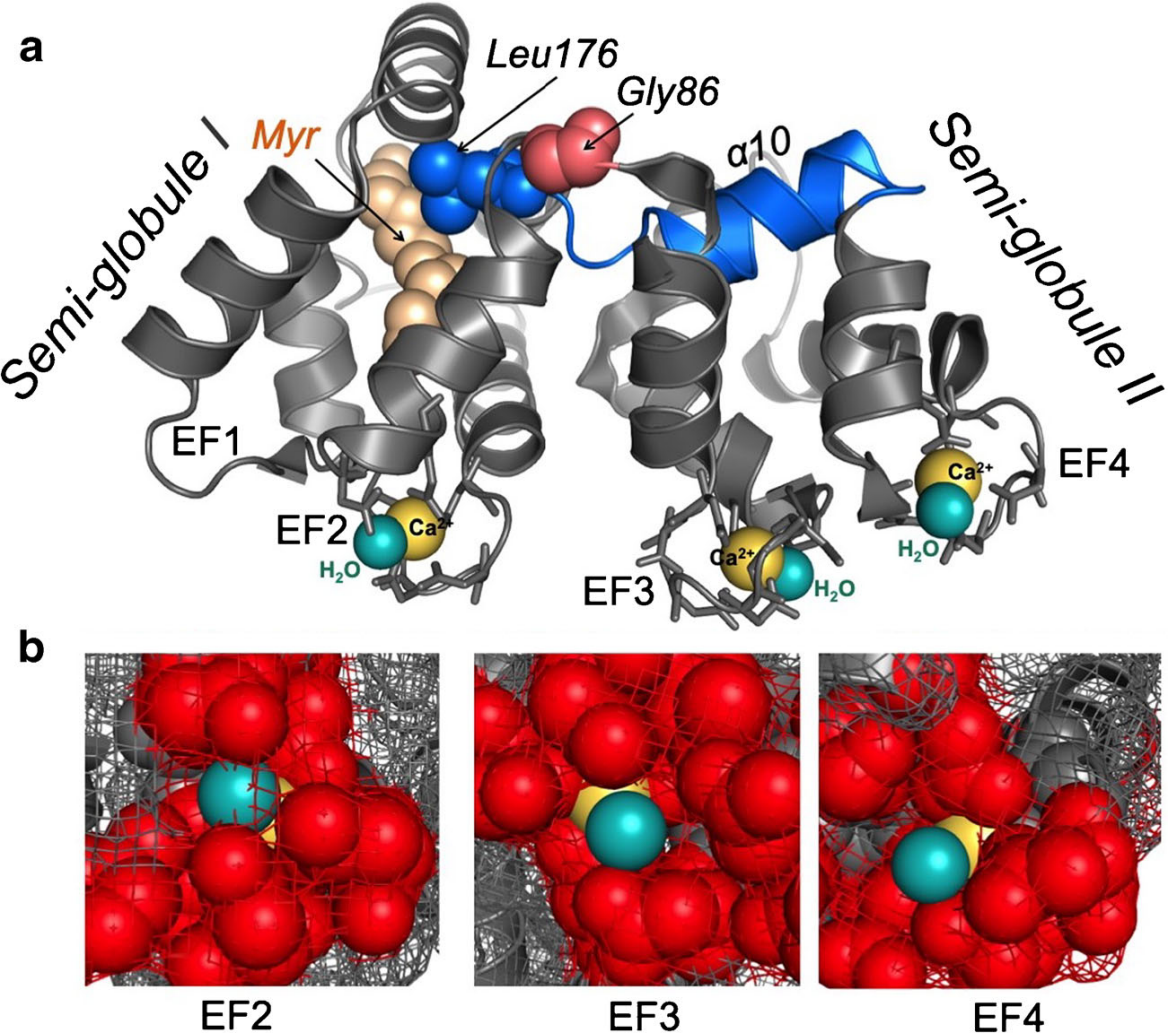
**a** Three-dimensional ribbon diagram of GCAP1 indicates the positions of Ca^2+^ (yellow) and H_2_O (light cyan) coordinated in three EF-hand loops; the calcium-myristoyl tug structure is highlighted in blue and the hinge Gly-86 between the two semi-globules is shown in pink. **b** Space-filled/mesh close-up diagram of the Ca^2+^:H_2_O position in EF-hand structures; red space-filled side chains in the loop, yellow Ca^2+^, cyan H_2_O (the models utilize the molecular coordinates of the GCAP1 crystal structure reported by Stephen et al. [111]

GCAPs have three active metal binding EF-hands: EF2, EF3, and EF4 (Figs. 2, 4). Ca^2+^ binding in different EF-hands has been studied by selectively inactivating other EF-hands using point mutations introduced in 12-residue EF-hand metal binding loops. Subsequent assessment of the individual EF-hand affinities for Ca^2+^ using the intrinsic GCAP1 tryptophan fluorescence spectra and titration using Ca^2+^ indicator dyes [83] gives a range of their *K*_dCa_ between ~26 and ~120 nM, being the lowest in EF2 (the highest-affinity site) and the highest in EF4 (the lower-affinity site). Another important observation from studying the Ca^2+^ sensitivity of individual EF-hands was that although binding of Ca^2+^ in EF2 does not require Ca^2+^ to occupy EF-hands 3 or 4, binding of the metal in EF4 strictly requires EF3 being liganded by Ca^2+^ [84, 87]. In other words, there are two Ca^2+^-sensor parts of GCAP1: one operates through EF2 and the other through a pair of EF-hands 3 and 4 acting in concert [84]. The high-resolution crystal structure of myristoylated GCAP1 in its Ca^2+^-bound state established by Stephen et al. [111] also helps to weigh the relevance of the apparent *K*_dCa_ values for Ca^2+^-binding in EF-hands to the structural organization of GCAP1 (Fig. 4). Overall, the Ca^2+^ binding affinities derived from the tryptophan fluorescence spectroscopy and titration using  Ca^2+^ indicator dyes [83] agrees quite well with the three-dimensional structure of GCAP1 EF-hands in a Ca^2+^-liganded state (Fig. 4). The three EF-hands in GCAP1 hold Ca^2+^ using a typical for EF-hands seven-dentate coordination in a 12-residue loop. Similarly to some EF-hands of this type [13, 14], a water molecule provides one of the essential hydrogen bonds coordinating Ca^2+^ in the loop. All three metal binding EF-hands in GCAP1 use water to hold Ca^2+^ in the loop, much like a “stopper” plugging the exit channel for the metal ion (Fig. 4). These water molecules are clearly visible in the crystal structure established by Stephen et al. [111]. The three-dimensional EF-hand structure reveals that the Ca^2+^:H_2_O pairs are better embedded into the EF2 than in the other two metal-binding EF-hands. Conversely, in EF4, the water molecule mediating coordination of Ca^2+^ is exposed near the very surface, indicating that the metal ion is held in the EF4 loop more flexibly than in EF2 (Fig. 4b).

Notably, the affinities of the individual EF-hands for metal ions estimated using isothermal titration calorimetry (ITC) [2, 60, 96] rank their relative affinities for Ca^2+^ that are different from those derived from the tryptophan fluorescence spectroscopy and are not consistent with the crystal structure of GCAP1 (Fig. 4). In addition, contrary to the apparent *K*_d_ determined by the fluorescence spectroscopy, some of the affinities estimated by ITC do not fit well with the physiological range of free Ca^2+^ in the outer segment. A likely reason for that is that the heat release for GCAPs in response to the binding metal does not present a simply exothermic or endothermic pattern, but includes a combination of both [60, 96]. When a complex kinetics of the heat release is simplified by formally applying one- or two-center binding models, it evidently skews the accuracy of measuring the individual EF-hand affinities for the metal ligand. In contrast, the binding isotherms derived from the tryptophan fluorescence directly reflect conformational changes in the protein that occur as a result of metal binding and are not affected by a complex pattern of the heat release and absorption. So even though ITC microcalorimetry can be very useful in some aspects of structural studies in GCAPs [2, 96], its usefulness for measuring specific parameters of metal binding should be evaluated with a great deal of caution.

As an important characteristic of the Ca^2+^-sensitive regulation of RetGC by GCAPs, the effect of Ca^2+^ is highly cooperative (the Hill coefficient ≥ 2) [53, 68, 69, 82, 89]. The isotherm for Ca^2+^ binding to a purified GCAP1 shows that the binding occurs within the same physiological range of Ca^2+^ as the regulation of RetGC, but surprisingly does not show cooperativity (the Hill coefficient of 1) [83]. It is therefore possible that in GCAPs bound to its target enzyme, the interaction between different EF-hands change to bind the metal in a cooperative manner.

Another important aspect of GCAP-mediated Ca^2+^-sensitive regulation of retinal guanylyl cyclase is that it is also highly sensitive to Mg^2+^[29, 82], because all metal binding EF-hands in GCAP also bind Mg^2+^ with submillimolar affinities [83, 84]. Although GCAPs, like other Ca^2+^ binding proteins, undergo conformational changes between different functional states, these conformational changes are rather subtle when compared to calmodulin or even to recoverin [62]. These conformational changes in GCAPs are not robust because GCAPs are liganded by divalent cations in both functional states [60, 62]. In the “activator” state of GCAP, Mg^2+^ almost fully substitutes Ca^2+^ in at least two and probably all three metal binding EF-hands [83, 84]. The affinity of GCAP1 for Mg^2+^ is ~1000-fold lower than for Ca^2+^ [82, 83], but the physiological concentrations of Mg^2+^ in the outer segment, near 1 mM [20], vastly exceed the ~20–50 nM Ca^2+^ concentration in illuminated photoreceptors [37, 121]. Therefore, when the free Ca^2+^ concentrations in the outer segment fall after illumination, Mg^2+^ can easily displace Ca^2+^ from EF-hands 3 and 4 (and even from EF2 in a substantial fraction of GCAP1) [82–84]. Preservation of the cation-bound state both in the dark and in the light is essential for the regulatory function of GCAP, because the apo-form of GCAP1 is a completely inactive protein that fails to bind with RetGC [84, 87]. To preserve the GCAP ability to bind to the target enzyme, a divalent cation, either Mg^2+^ or Ca^2+^, must occupy both EF-hands 2 and -3 [84, 87]. Hence, the replacement of Ca^2+^ by Mg^2+^ in the light enables GCAPs to stay in complex with RetGC even after the CNG channels become closed and the concentration of intracellular free Ca^2+^ in light-adapted photoreceptors falls. Mg^2+^ binding also plays a crucial role in adjusting the Ca^2+^ sensitivity of GCAPs to the physiological range of free Ca^2+^ in the outer segment. The exact concentrations of Mg^2+^ in mammalian photoreceptors have never been directly measured, but the Ca^2+^ sensitivity of RetGC controlled by GCAP1 and GCAP2 in mouse photoreceptors best fits into the physiological range of free Ca^2+^ at the free Mg^2+^ concentrations near 1 mM [83], close to the physiological levels of Mg^2+^ measured in rods of lower vertebrates [20]. If GCAP EF-hands were unable to bind Mg^2+^, it would effectively undercut the dynamic range of RetGC regulation: when Mg^2+^ concentrations are reduced, the inhibition of the RetGC/GCAP complex by Ca^2+^ becomes too sensitive, and the cyclase activity decelerates well before the free Ca^2+^ reaches its normal dark-adapted level. Conversely, increase of GCAP affinity for Mg^2+^ versus Ca^2+^ would require much higher concentrations of Ca^2+^ to decelerate the cyclase. As we will further discuss in this article, such a low sensitivity of the cyclase to the inhibition by Ca^2+^ abnormally elevates Ca^2+^ influx through CNG channels and provokes photoreceptor death [105]. Evidently, the structures of EF-hands in GCAPs evolved to achieve the optimal balance between their affinities for Mg^2+^ versus Ca^2+^ and thus to optimize the dynamic range of the cyclase regulation within the physiological ranges of the two cations in photoreceptors [29].

**Fig. 5:**
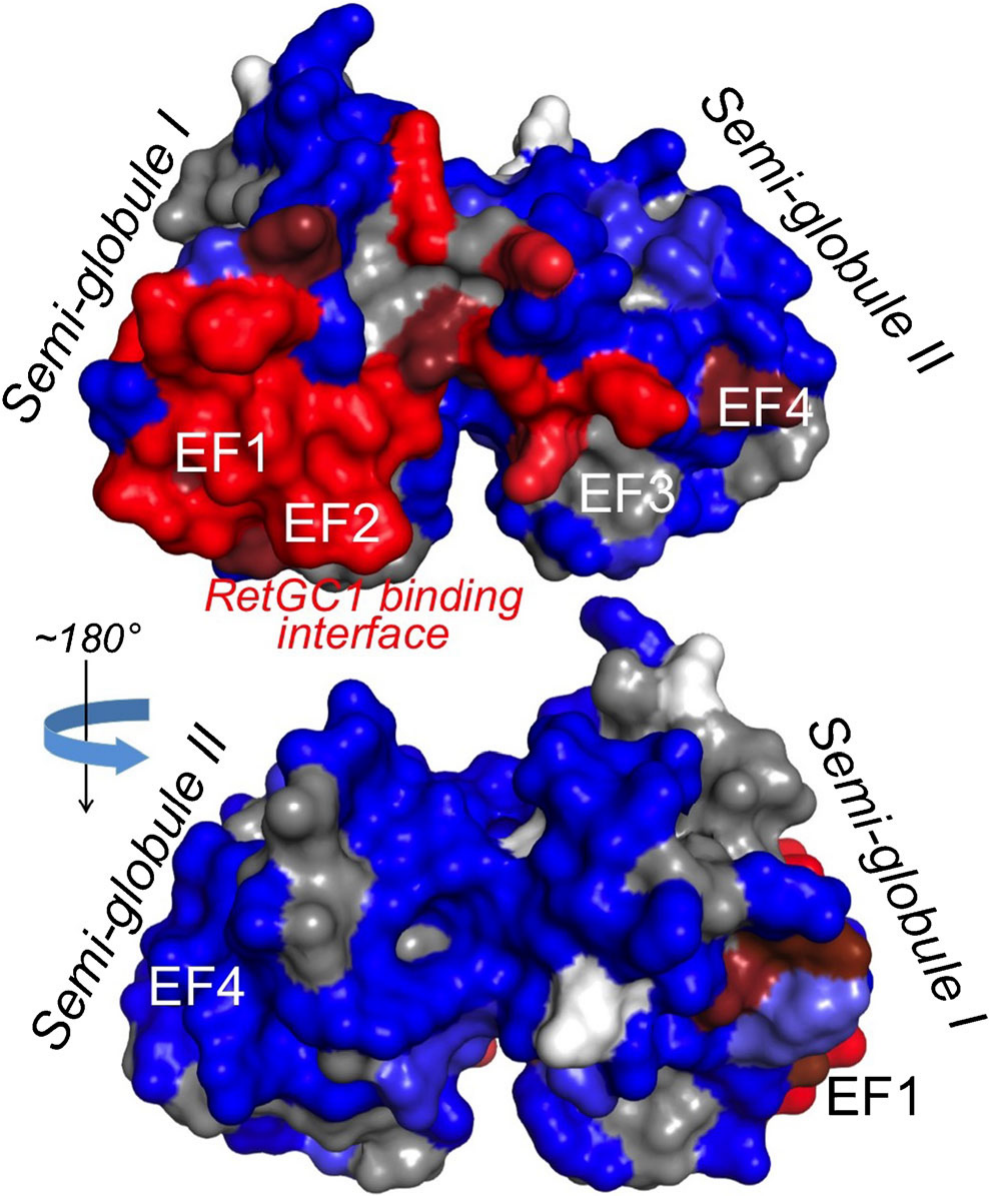
RetGC-binding interface in GCAP1. The surface-exposed residues critical for the regulatory binding to RetGC are highlighted in red, and the non-essential residues are highlighted in blue [92]. The main part of the binding interface on GCAP1 occupies EF-hand 1 and 2 in semi-globule I, whereas the most critical Ca^2+^ sensor function belongs to the semi-globule II containing EF-hands 3 and 4

Whereas holding Mg^2+^ or Ca^2+^ in EF2 and EF3 allows GCAPs to always bind its target enzyme [58, 87], binding and release of Ca^2+^ in EF4 are most critical for switching GCAPs between its RetGC-activator and RetGC-inhibitor states. Inactivation of the cation binding in EF4 locks GCAPs in a perpetual activator state, accelerating RetGC activity even at such free Ca^2+^ concentrations that by far exceed the normal physiological levels in the dark [22, 83, 87]. Mg^2+^ binding in EF4 is not essential for the ability of GCAPs to activate RetGC, but helps to adjust its Ca^2+^ sensitivity to the physiological range of Ca^2+^ [82]. Structural changes in EF4 have a major regulatory effect even though the main part of the RetGC-binding interface in GCAP1 is located in the lobe comprised by the N-terminal EF-hands 1 and 2 [92]. This happens because the relatively subtle movement of the exiting helix in EF4 affects the GCAP1 fold on the other side of the molecule [63] (Fig. 3) and dimerization state of GCAP1 [12, 64]. Extensive site-directed mutagenesis of GCAP2 [32, 78] and especially GCAP1 [92] identified the cyclase binding interface of GCAP as a compact patch of the residues located on one side of the molecule and occupying large portions of EF1, -2, and a part of the entering helix of EF3 [92] (Fig. 5). Notably, the loop motif in GCAP EF-hand 1 lacks some side chains required for effective coordination of Ca^2+^ [13, 14, 57, 61]. Evidently, in the course of the GCAPs’ evolution, their N-terminal EF-hand traded its original function of binding metal for a new function: binding the target enzyme [32, 42, 92].

In GCAP1, a structural link between the C-proximal EF4 and the N-terminal lobe occurs through a “calcium-myristoyl tug” [91], which directly links the two parts of the molecule: one responsible for recognition of the target enzyme and the other acting as the Ca^2+^ sensor for the change of its functional state (Fig. 3). The main portion of the GCAP1 molecule that creates the cyclase-binding interface also contains the N-myristoyl residue embedded in the semi-globule I, between EF-hands 1 and -2 (Figs. 3, 4). The molecular dynamic of this part of the molecule also indicates that these residues are most likely to allow GCAP1 to change its interactions with the cyclase in response to the change of the metal-liganded state [103]. As the exiting helix of EF4 continues to the C-terminal helix 10 extending to the opposite side of GCAP1, Leu-176 directly contacts the fatty acyl moiety (Figs. 3, 4) [111]. This link enables the tug action between EF4, the main Ca^2+^ sensor part in the cyclase regulation, and the cyclase-binding interface [91]. The pull-push action of the tug [70, 71, 90, 91] tunes EF4 affinity for Ca^2+^, adjusting the Ca^2+^ sensitivity of the GCAP1 activator-to-inhibitor conversion and its affinity for RetGC at the same time [90, 91]. Artificial changes in the length and/or composition of the tug can further increase GCAP1 affinity for the cyclase, but only at the expense of reducing its affinity for Ca^2+^, and vice versa [91]. Apparently, the calcium-myristoyl tug has evolved as yet another fine adjustment of the GCAP1 structure to its role as a Ca^2+^ sensor in regulation of RetGC in physiological conditions optimal for the photoreceptor function.

## The roles of GCAPs in shaping photoresponse

Ca^2+^-sensitive regulation of RetGC is the predominant component of the Ca^2+^ feedback in photoreceptors [101]. The kinetics of rod recovery is limited primarily by the rate of rhodopsin-transducin-PDE6 cascade deactivation, rather than by the replenishment of cGMP by guanylyl cyclase [38, 55]. The cyclase activity not only does not rate-limit the recovery of normal rods, but even when overexpression of RGS9 stimulates deactivation of transduction and makes rod responses recover more quickly, the activity of RetGC apparently remains high enough not to limit the accelerated responses [55]. However, the high activity of RetGC during the photoreceptor recovery only becomes possible after its robust activation by GCAPs via negative Ca^2+^ feedback. In the absence of GCAPs, rods and cones retain their general ability to hyperpolarize in the light, but they recover from excitation much more slowly than normal [15, 72].

GCAP1 and GCAP2 are the two ubiquitous isoforms of GCAPs present in all vertebrate species tested to date. They are also the only two isoforms encoded by the mouse genome [72]. When the tail-to-tail adjacent *Guca1a* and *Guca1b* mouse genes, coding respectively for GCAP1 and GCAP2, are simultaneously disrupted by a gene knockout construct, rod dim flash responses grow very large and recover very slowly [15, 72]. So RetGC, even without its activator proteins, can restore the permeability of the CNG channels, but the rods operating only on the basal cyclase activity fail to restrict the amplitude of a single-photon response and to recover in a timely fashion, thus losing the temporal resolution of their responses and becoming hypersensitive to light. Cones lacking GCAP1 and GCAP2 also prolong their recovery phase, albeit less dramatically than rods [104]. The responses in GCAP-deficient cones still remain much faster than in rods, possibly because cones have higher RetGC content than rods [11, 25, 123].

**Fig. 6:**
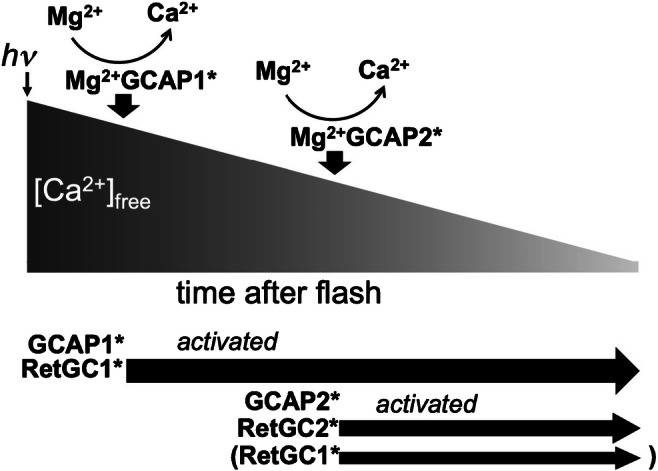
Sequential activation of RetGC isozymes by GCAPs in rods via Ca^2+^ feedback. GCAP1 has lower affinity for Ca^2+^ and therefore activates RetGC1 soon after the high dark Ca^2+^ levels in rods start to decline at the beginning of the photoresponse. The early activation of RetGC1 restricts the amplitude of a photon response. GCAP2 has higher affinity for Ca^2+^ and converts to the Mg^2+^-liganded state only after the free Ca^2+^ levels further decline in mid-phase of the response; it then activates the ancillary RetGC2 (and possibly a fraction of RetGC1 unoccupied by GCAP1) [68, 69]

Rods express both GCAP1 and GCAP2, whereas cones almost exclusively express GCAP1 [68, 69, 123]. Rods need both GCAP1 and GCAP2 to properly shape their responses to a dim flash. Although very similar in their main structural characteristics, the two Ca^2+^ sensors have slightly different operational ranges: GCAP1 is less and GCAP2 is more sensitive to Ca^2+^ [41, 89]. Under physiological conditions, RetGC activity stimulated by GCAP1 requires higher Ca^2+^ concentrations to decelerate it (IC_50_ ~130 nM) [68, 69, 89]. Conversely, deceleration of the cyclase stimulated by GCAP2 (IC_50_ ~50–60 nM) occurs at free Ca^2+^ concentrations lower than in GCAP1 [68, 69, 89]. Individual GCAP gene knockouts [68, 69] indicate that the two GCAPs activate RetGC in response to light by acting in a sequential mode (Fig. 6). GCAP1 is the first-response sensor that starts to activate RetGC as soon as the intracellular Ca^2+^ begins to drop following the activation of the phototransduction cascade. RetGC activity stimulated by GCAP1 restricts the amplitude of the dim flash response, by not allowing rods to close an excessive number of channels after absorbing a small number of photons, which helps to extend the dynamic range of rod responses. The free Ca^2+^ levels have to fall deeper in mid-phase of the response, before GCAP2-stimulated RetGC activity can kick in and accelerate the recovery. The amplitudes of dim-flash rod responses in GCAP1^-/-^ mice, where upregulated GCAP2 takes over the RetGC activation, grow larger than normal, albeit not as large as in GCAP1/GCAP2 double-knockouts, and reach the peak later than normal, yet their recovery from the excitation occurs with a near-normal kinetics [69]. Conversely, in GCAP2^-/-^ mice, the amplitude and the time to peak of rod responses remain similar to normal, but they recover more slowly [68].

It is also worth mentioning that the term “relay” that has been used to describe the sequential activation of the cyclase by GCAPs [51, 69] is somewhat inaccurate, because the activation of the cyclase by GCAP1 continues even after Ca^2+^ falls to the levels sufficient for activation by GCAP2. Contrary to the “relay” fashion, the two GCAPs do not replace each other; rather, the fraction of the cyclase activity stimulated by GCAP2 in mid-phase of the response adds to the activity of the cyclase pool stimulated by GCAP1 in the earlier phase of the response (Fig. 6).

In cones, the need for sequential activation of RetGC by GCAPs is less evident, because GCAP1 heavily dominates regulation of RetGC by Ca^2+^ [123]. However, GCAP2 can accelerate the recovery in cones lacking GCAP1, making it faster than in cones lacking both GCAP1 and GCAP2 altogether [118].

## Regulatory properties of retinal guanylyl cyclase

Retinal membrane guanylyl cyclase (RetGC) regulated in photoreceptors by GCAPs exists as two isozymes, RetGC1 and RetGC2 [25, 66, 125]. In mouse and rat genomes, *Gucy2e* and *Gucy2f* genes code for the respective orthologs, GC-E and GC-F, of human RetGC1 and RetGC2 [126]. The suggested elsewhere use of the name GC-E for human RetGC1, in our opinion, creates unnecessary confusion, because RetGC1 in humans is coded by *GUCY2D* gene, not by *GUCY2E*, which in primates codes for a pseudogene homologous to the olfactory guanylyl cyclase (conversely, in rodents the *Gucy2d* gene codes for an olfactory cyclase). This is why we hereafter prefer, for the sake of clarity, to keep the original designations for the two isozymes, RetGC1 and RetGC2 [25, 66] for all species, regardless of the names of their respective coding genes.

The joined activity of RetGC1 and RetGC2 presents the only source of cGMP in phototransduction. Ca^2+^-sensitive cGMP production is undetectable in mice lacking both isozymes, and their retinas do not respond to light [11, 89]. However, RetGC1 and RetGC2 do not contribute equally to cGMP synthesis in photoreceptors, and they also show different distributions between rods and cones. RetGC1 is the main isozyme of the cyclase, present in both rods and cones [11, 89, 123]. In rods, RetGC1 is the predominant form of the cyclase, responsible for the bulk (≥70%) of the cGMP production in outer segment, and RetGC2 isozyme acts as the ancillary component, producing < 30% of the total cGMP [89]. RetGC2 is virtually undetectable in cones [123], whereas RetGC1 is expressed in cones better than in rods [11, 25, 123]. Consequently, rod mass responses (as detected by scotopic electroretinography (ERG)) to dim flashes strongly diminish in mice lacking RetGC1 [127]. At the same time, some RetGC1-deficient rods respond to light with near-normal amplitude and with only a slight delay in the onset of the recovery, whereas RetGC1-deficient mouse cones become completely non-responsive to light [127].

**Fig. 7:**
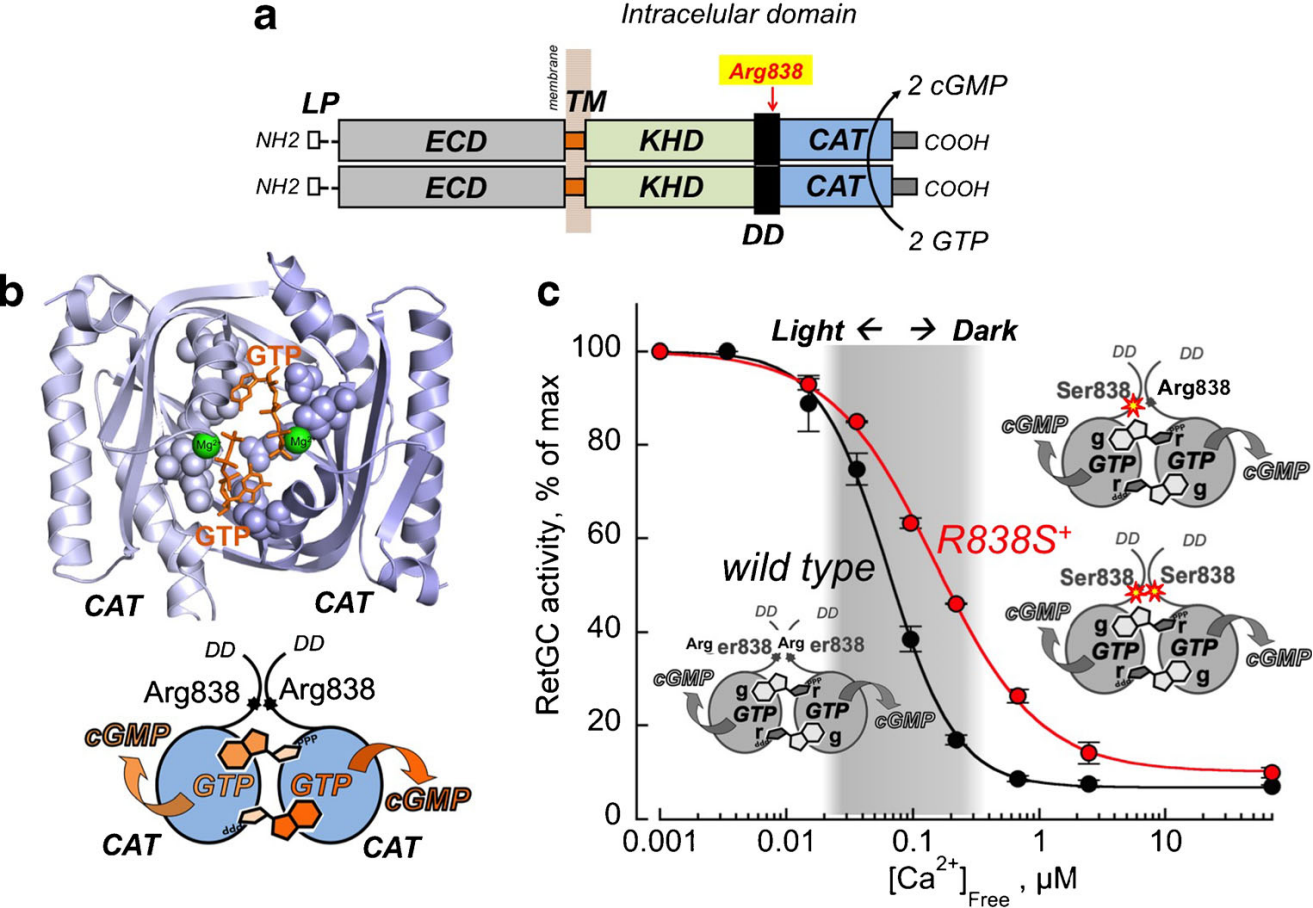
**a** The diagram of RetGC1 homodimer. Each *GUCY2D*–coded polypeptide includes the N-terminal leader peptide (LP), “extracellular” domain (ECD), transmembrane region (TM), kinase homology domain (KHD), dimerization domain (DD), and catalytic domain (CAT) [36, 66]. Two RetGC1 subunits in the homodimer catalyze GTP-to-cGMP conversion by the two catalytic domains forming a single active site; the arrow indicates the position of Arg838 frequently substituted in *GUCY2D* CORD6 alleles [40, 106]. **b** Top panel. The three-dimensional model of the dimerized RetGC1 catalytic domains making a single active site for binding simultaneously two Mg^2+^ GTP substrate molecules (Liu et al. [65]). The two GTP molecules are highlighted in orange and the two Mg^2+^ counterions in green. The schematic in the bottom panel illustrates that each of the two GTP molecules in the active site is simultaneously held by both RetGC1 subunits; none of the subunits can bind GTP on its own. **c** CORD6 mutation, Arg838Ser, reduces Ca^2+^ sensitivity of both homodimer Ser838:Ser838 [86, 102] and heterodimer Arg8383:Ser838s [98]; therefore, in transgenic mice expressing both wild type and Arg838Ser RetGC1 (red), RetGC remains active outside the physiological range of free Ca^2+^ in the dark (shaded in gray) [30, 105] (the data in the graph are from Dizhoor et al. (2016), article in *Journal of Biological Chemistry* [30])

In their functional state, RetGC1 and RetGC2 are homodimers [65, 124]. The RetGC1:RetGC2 heterodimers can be produced in cultured cells, but do not exist in living photoreceptors in vivo [124]. RetGC1 and RetGC2 are closely homologous to each other and share similar domain structure with other membrane receptor guanylyl cyclases [36, 66]. A 120-kDa RetGC polypeptide (Fig. 7a) starts with a ~5-kDa leader peptide (cut-off from a mature protein), followed by a 43-kDa “extracellular” domain. In the photoreceptor outer segment, the extracellular domain of the cyclase is primarily exposed in the intradiskal space at the periphery of the disk [76]. Therefore, in cones, where all photoreceptor disks are invaginations of the plasma membrane, that portion of the cyclase is a true extracellular part of the enzyme, but in rods, where the mature photoreceptor disks are themselves completely inside the outer segment, it is not truly “extracellular.” The ~70-kDa cytoplasmic portion of RetGC includes two major domains: a proximal to the membrane kinase-homology domain and proximal to the C-terminus catalytic domain [36, 66]. These two intracellular domains are connected to each other via a short coiled-coil dimerization domain [36, 66, 102].

The three-dimensional structure of RetGC has not been determined, but the modeled structure of its catalytic domain, built based on the close homology to that of the established structure of adenylyl cyclase [65], gives a reliable approximation, verified by functional studies and site-directed mutagenesis [102, 114]. The active site of the cyclase, which catalyzes conversion of the GTP to cGMP and inorganic pyrophosphate, binds two Mg^2+^ GTP substrate molecules at the same time, with each substrate molecule simultaneously held by two opposing subunits in the homodimer [65, 114] (Fig. 7b).

The basal activity of RetGC in the absence of GCAPs is detectable, but rather low [72, 89]. GCAPs bind RetGC in both cation-liganded forms, Mg^2+^ in the light and Ca^2+^ in the dark [29, 58], switching the cyclase between the respective “activated” and “inhibited” states. Mg^2+^ GCAP increases the *V*_max_ of the reaction catalyzed by RetGC with a moderate effect on the *K*_mGTP_ [25, 89], whereas Ca^2+^ GCAP decelerates the cyclase activity even to below its basal level in washed outer segment membranes [22, 27]. Notably, Mg^2+^ is required for catalytic activity of RetGC as a part of Mg^2+^ GTP substrate, but this does not relate to the Mg^2+^ sensitivity of the Ca^2+^-dependent regulation by GCAPs discussed in the previous section [82]. Based on studying mouse gene-knockout models, the Ca^2+^ sensitivity of RetGC isozyme is defined solely by the Mg^2+^ versus Ca^2+^-affinities of the two main GCAP isoforms [89]. Both GCAP1 and GCAP2 stimulate RetGC1 and RetGC2 in vitro [89], but not in vivo. Biochemical and physiological studies conducted in mouse models expressing different combinations of GCAP isoforms and RetGC isozymes demonstrate that GCAP2 can activate both RetGC1 and RetGC2, whereas GCAP1 almost exclusively regulates RetGC1 [80]. Therefore, the sequential activation of the RetGC by the two GCAPs via Ca^2+^ feedback can be further extended to a sequential activation of the two RetGC isozymes (Fig. 6): GCAP1 starts activating RetGC1 in the early phase of the photoresponse, and then GCAP2 adds the ancillary activation of RetGC2 (and possibly of a RetGC1 fraction unoccupied by GCAP1) in the mid-phase of the response.

Considering the specificity of GCAPs in the regulation of RetGC isozymes, it is important to emphasize that the physiological data assessing the interactions between the endogenous RetGC1 isozyme and GCAP isoforms in vivo using gene-knockout and transgenic mouse models unambiguously demonstrate that GCAP2 can effectively stimulate RetGC1, whereas GCAP1 (either endogenous murine or transgenically expressed bovine) does not significantly stimulate RetGC2 in vivo [80]. A controversy in the field remains about how effectively GCAP2 can activate human RetGC1 [21, 98]. It appears that GCAPs of different species expressed in *Escherichia coli* can be sensitive to the process of renaturation after extraction from the inclusion bodies, because a human GCAP2 refolded from guanidine chloride used as denaturing agent is unable to stimulate human RetGC1 in vitro [21], whereas the same isoform purified using urea-extraction activates human RetGC1, albeit with an apparent affinity lower than GCAP1 [98]. Obviously, it is the physiology that should ultimately elucidate the role of GCAPs. Notably, a murine GCAP1 efficiently activates RetGC2 in vitro [89], but not in vivo [80]. The same (or the opposite) could conceivably be true for other species. In our opinion, unless directly supported by physiological or clinical evidence, conclusions from in vitro studies exclusively utilizing recombinant proteins or computer stimulations should be taken with a “grain of salt” when it comes to such a kinetically complex sensory system as photoreceptors. Loss-of-function mutations in human GCAP1 and GCAP2 could shed some light on the uncertainty. Yet, as we discuss later in this review (the “Abnormal Ca^2+^ feedback leads to photoreceptor death” section), no recessive loss-of-function mutations in GCAP1 (or GCAP2) affecting human vision were identified to date, in contrast to the multiple recessive mutations in RetGC1, causing *GUCY2D* retinal blindness. This may indirectly indicate that the both GCAPs activate human RetGC1 in vivo; otherwise, a loss of GCAP1 function alone would have likely resulted in finding vision disorders similar to the recessive *GUCY2D* blindness.

## The molecular interactions in the RetGC:GCAP complex

The structure of the RetGC, let alone the RetGC:GCAP complex, remains unknown, and most of our current knowledge about the regulatory interactions between the cyclase and its Ca^2+^ sensor protein comes from site-directed mutagenesis and functional studies in vitro. The location of the binding interface for the cyclase on GCAP1 has been determined by probing the entire surface of GCAP1 by point mutations (Fig. 5) [92]. Identification of the GCAP-binding interface on RetGC, however, still presents a major challenge, largely because the cyclase cannot be isolated from the membranes in a soluble form without losing its regulation by GCAPs [50] and because the catalytic domain, the only part of the cyclase for which there is a reliable three-dimensional model [65], is not the part of the enzyme regulated by GCAPs [93, 94]. When a catalytic domain from a GCAP-insensitive hormone-receptor cyclase, human GUCY2A, replaces the catalytic domain of RetGC, the resultant chimera still retains sensitivity to regulation by GCAPs. In contrast, replacing the kinase homology or dimerization domains completely eliminates the ability of such chimeras to bind GCAP1 and GCAP2. Point mutations in the RetGC1 kinase homology domain and dimerization domain can also completely disrupt GCAP binding [93, 94]. The importance of the dimerization domain in RetGC regulation is further evidenced by the mutations causing congenital blindness, as described in the following section.

## Abnormal Ca^2+^ feedback leads to photoreceptor death

Multiple mutations in RetGC1 cause various forms of congenital blindness (reviewed in a great detail in a recent publication by Sharon and colleagues [106]). Most of the mutations frequently found in *GUCY2D* cause a recessive blindness from birth, called Leber’s congenital amaurosis (LCA) [45, 112]. These mutations can prematurely terminate the RetGC1 polypeptide, cause a frame-shift, or inactivate the cyclase by single-residue substitutions. As the result of such mutations, cones typically become non-responsive, whereas rudimentary rod vision can sometimes be detected [45]. Although not identical to the phenotype of the human disease, the changes in retinal physiology observed in mice lacking RetGC1 [127], to some extent, resemble those in *GUCY2D* LCA [45]. One of the most important observations in both cases is that the blindness caused by the recessive mutations in RetGC1 does not entail massive photoreceptor degeneration [45]. This opens a possibility of applying gene therapy to restore vision by delivering the normal RetGC1 gene in the diseased photoreceptor using viral vectors [45, 46]. More recently, another form of congenital blindness—congenital stationary night blindness (CSNB)—was linked to recessive mutations in *GUCY2D* [113]. In this form of blindness, night vision (rod function) becomes disabled, but daylight vision (cone function) remains virtually unaffected. Paradoxically, CSNB alleles [113] in these cases code for inactive or nearly inactive RetGC1 [98], much as in *GUCY2D* LCA. It remains unknown at this point why these mutations cause CSNB rather than LCA, but the cause of *GUCY2D*-linked CSNB and LCA evidently relates to the lack of the RetGC1 activity rather than abnormality in its Ca^2+^ sensitive regulation.

In contrast to LCA and CSNB, *GUCY2D* mutations that cause deregulation of the cyclase by Ca^2+^ feedback do not disable photoreceptor function, but rather cause premature death of well-responsive photoreceptors. Multiple mutations in RetGC1 and GCAPs causing this type of blindness have been excellently summarized in some comprehensive reviews [40, 106]; so in our present review, we will only briefly reflect on the most essential elements of the cyclase regulation by GCAPs affected by the dominant mutations that cause photoreceptor degenerations. Mutations of this type have the dominant phenotype because they decrease RetGC sensitivity to deceleration by Ca^2+^ (Fig. 7). The shift in Ca^2+^ sensitivity of the cyclase beyond the normal free Ca^2+^ levels in the dark can result from mutations in the cyclase itself or in GCAP1, its main regulator in photoreceptors [40, 106].

Mutations most prominently affecting Ca^2+^-sensitive regulation of RetGC1 by GCAPs have been found in the cyclase dimerization domain, where Arg-838 (Fig. 7a) becomes replaced by Ser, His, Cys, or Pro [49, 40, 102, 115]. These mutations cause dominant cone-rod dystrophy (CORD6, or adCORD), a form of degenerative blindness that typically starts at a young age as dystrophy of cones in the central retina and then progresses to the periphery of the retina involving rods [40]. Replication of the CORD6-linked Arg-838 substitutions in a recombinant RetGC1 causes a prominent right-shift in the Ca^2+^ sensitivity of the cyclase regulation by GCAPs in vitro [102, 114, 115] and in vivo in transgenic mice [30, 105] (Fig. 7c). Whereas it may appear surprising that mutations in the cyclase, rather than in its Ca^2+^ sensor protein, cause the abnormal Ca^2+^ sensitivity of its regulation, there is a reasonable explanation for it, which is that the mutant RetGC1 gains much higher affinity for the Mg^2+^ GCAPs than for Ca^2+^ GCAP [30, 102, 114]. As the Mg^2+^ and Ca^2+^ GCAP1 compete for the cyclase, the increased affinity for the activator form of the sensor protein requires converting a larger fraction of GCAP1 into a Ca^2+^-liganded state to outcompete the remaining Ca^2+^-free/Mg^2+^-liganded GCAP1 from the complex and decelerate the cyclase. Another possible explanation is that by increasing GCAP affinity for the cyclase, the mutations in Arg838 directly reduce the GCAP1 affinity for Ca^2+^ when in the complex with the mutant cyclase [114]. Distinguishing between the two of these related to each other possibilities would, however, require isolation of the functional RetGC1/GCAP1 complex, which is currently not achievable due to the instability of such a complex in detergents [50].

The net result of the mutations in Arg-838 is the increase of Ca^2+^ influx in the photoreceptor outer segment. In normal photoreceptors, cGMP production by RetGC becomes decelerated when only a small fraction of the CNG channels in the outer segment plasma membrane opens and restores the influx of Ca^2+^, sufficient to convert GCAP into its 'RetGC1 inhibitor' state. Deceleration of the cyclase prevents excessive opening of the CNG channels and a further increase of the Ca^2+^ influx into the outer segment. In the case of Arg-838 substitutions, the cyclase would continue to produce cGMP at the concentrations of Ca^2+^ exceeding the normal levels in the dark and to open a larger than normal fraction of CNG channels before the increased Ca^2+^ influx becomes high enough to finally decelerate RetGC. High Ca^2+^ and cGMP concentrations can then trigger the apoptotic processes in photoreceptors [123], thereby causing their degeneration. Study of the in vivo models supports this scenario. In transgenic mice expressing Arg838Ser RetGC1 [30], the Ca^2+^ sensitivity of RetGC regulation becomes shifted toward higher Ca^2+^ concentrations (Fig. 7c), which increases cGMP-gated current through CNG channels and elevates Ca^2+^ influx in the outer segment [105]. Importantly, deletion of GCAPs completely rescues degeneration of the Arg838Ser RetGC1-expressing rods, thus indicating that the mutated cyclase causes their death only as a result of the abnormal Ca^2+^ feedback regulation and confirming that CORD6 degeneration linked to the substitutions of Arg-838 is a “phototransduction disease” [105].

Mutations in GCAP1 (*GUCA1A*) typically cause dominant cone- or cone-rod degenerations [40, 52, 106] by preventing GCAP1 from acquiring its Ca^2+^-liganded conformation at normal Ca^2+^ concentrations in the dark. Different molecular mechanisms can underpin this effect in the case of GCAP1: (1) The disease-causing mutations (such as in Glu155Gly GCAP1 [120]) can directly affect Ca^2+^ binding in EF4, the EF-hand most critical for converting GCAP1 in the “RetGC inhibitor” state [84, 87]. (2) They can also affect EF4 indirectly, through the neighboring EF3. The two EF-hands act in concert, such that high-affinity Ca^2+^ binding in EF4 is only possible when EF3 binds Ca^2+^ [83]. Consequently, the reduced affinity for Ca^2+^ in EF3 (such as in Tyr99Cys GCAP1 or Glu11Val [27, 71, 108]) can also convert GCAP1 into a Ca^2+^-insensitive activator of RetGC. (3) Some mutations in GCAP1 can also affect its affinity for Ca^2+^ indirectly by changing the intermolecular interactions outside the immediate helix-loop-helix EF-hand domains. For example, the Leu176Phe substitution, originally used to identify and probe the calcium-myristoyl tug in GCAP1 in vitro [91], was subsequently found to cause severe dominant retinopathy in human patients [119]. This substitution strongly reduces the affinity for Ca^2+^ in EF4 via a push-pull action between the exiting helix of EF4 and the myristoyl residue inside the N-terminal semi-globule of GCAP1 [91, 119]. It also increases the Mg^2+^ GCAP1 affinity for RetGC1 [91], making it more difficult for Ca^2+^-liganded GCAP1 produced by the normal *GUCA1A* allele to compete with the Leu176Phe GCAP1. (4) Another example of the indirect effect of mutations in GCAP1 on its Ca^2+^ sensitivity was reported recently in a case of a dominant retinopathy caused by Gly86Arg substitution in a “hinge” region connecting the two lobes of GCAP1 [2, 96]. Fluorescence spectroscopy and EPR indicate that the two semi-globules in GCAP molecule evidently move around the “hinge” Gly (Fig. 4a), either in a twisted or a piston-like motion, when EF4 in GCAP1 binds and releases Ca^2+^ [54, 83, 109]. The Gly86Arg GCAP1 shows lower affinity for Ca^2+^ and higher affinity for RetGC [96], most likely because the altered flexibility of the “hinge” creates hindrance for changing the GCAP1 conformation into the “inhibitor” state, more effectively shifting it in the “activator” conformation [2, 83].

Much as in the case of the Arg838 substitutions in RetGC1, the net result of mutations in GCAP1 linked to the dominant retinopathies is the rise in intracellular free Ca^2+^. A large fraction of cGMP-gated channels open, and free Ca^2+^ concentrations rise, in rod outer segments of transgenic mice that express Tyr99Cys or Glu155Gly GCAP1 under control of a rhodopsin promoter [79, 122]. These transgenic models also demonstrate that the mutated GCAP1 shifts Ca^2+^ sensitivity of cGMP production in photoreceptors preferentially through the abnormal activation of the RetGC1 isozyme [80]. Consequently, deletion of RetGC1, but not RetGC2, dramatically rescues degeneration of rods harboring Tyr99Cys or Glu155Gly GCAP1 [80]. The abnormal activation of the cyclase by Ca^2+^-insensitive GCAP1 mutants leads to photoreceptor death by affecting the photoreceptors in their dark-adapted state, when the suppressed activity of PDE6 cannot effectively compensate for the accelerated activity of the cyclase. Artificial activation of PDE6 in the dark by expressing constitutively activated Gly90Asp rhodopsin [28, 107] dramatically rescues rods that express Tyr99Cys or Glu155Gly GCAP1 [122]. Rods in that case become partially “light-adapted” in the dark and more effectively hydrolyze cGMP produced by the deregulated cyclase, thus diminishing the fraction of the open CNG channels and reducing the abnormally high influx of Ca^2+^ in the dark.

It also worth noting that unlike mutations in RetGC1 that cause various recessive and dominant forms of blindness, all mutations identified in GCAP1 are, to our knowledge, dominant gain-of-function mutations [40, 106]. A likely reason why recessive mutations in GCAP1 causing loss of vision have not been reported to date is that a loss of GCAP1 or GCAP2 alone has a relatively modest effect on rod and cone physiology, largely compensated for by the presence (or even upregulation) of the other remaining isoform [69, 117]. Unless both GCAPs are inactivated simultaneously [15, 72], the relatively subtle changes in the kinetics of rod responses caused by the deficiency of a single GCAP isoform would conceivably only slightly affect rod (night) vision, not necessarily noticeable under the normal conditions of illumination.

## RD3 protein regulates retinal guanylyl cyclase and protects photoreceptors from degeneration

Adding more complexity to the control of RetGC activity in photoreceptors, the role of RD3, another regulator protein but not a Ca^2+^ sensor, has emerged from more recent studies [74]. RD3 was identified as a product of a recessive gene allele causing retinal degeneration-3 (*rd3*) in mice, hence the name of the protein. The loss of photoreceptors in *rd3* mice is distinct from various types of retinal degenerations in other mouse lines [18, 19]. At first, the *rd3* photoreceptors display near-normal morphology and abundance in the retina, but soon after completing their differentiation, they begin to degenerate. The exact rate of the degeneration varies depending on the particular background strain, but typically *rd3* rods and cones disappear within several months [34]. ERG photoresponses of *rd3* rods and cones are strongly suppressed [34], even prior to their en masse degeneration [95]. The cause of the degeneration is a nonsense mutation that truncates half of a 23-kDa RD3 polypeptide [34]. In a similar manner, mutations that truncate a human RD3 cause a recessive blindness from birth, Leber’s congenital amaurosis type 12 (LCA12) [34]. A long frame-shift in RD3 sequence also produces a canine retinal dysplasia [56].

RD3 has a dual function in photoreceptor physiology. Firstly, photoreceptors require RD3 in order to properly accumulate RetGC in the outer segment. There are indications that RD3 can be directly involved in the process of RetGC relocation from the photoreceptor inner segment to the outer segment [10, 131]. The content of RetGC1 and RetGC2 in *rd3* mice declines at least tenfold [10, 95, 99]. The much lower than normal levels of RetGC activity can well explain the very early photoreceptor dysfunction in *rd3* mice, resulting in a dramatic reduction of rod and cone ERG responses [95]. Gene therapy using an adenoviral vector to deliver the normal RD3 cDNA rescues *rd3* photoreceptors from degeneration and dysfunction [73].

However, the lower guanylyl cyclase activity in *rd3* photoreceptors per se is not the actual reason why they undergo rapid degeneration. Despite being significantly reduced, RetGC activity in *rd3* retinas remains detectable and retains regulation by Ca^2+^ and GCAPs [95]. Furthermore, rods and cones in young *rd3* mice still respond to light, albeit with much lower amplitudes of the ERG a-wave [31, 95]. Evidently, *rd3* photoreceptors still produce enough cGMP to support at least their rudimentary physiological responses. In a sharp contrast to that, in mice devoid of all RetGC activity by deletion of both RetGC1 and RetGC2 genes, rods and cones are completely unable to produce cGMP for phototransduction [11, 89] and fail to respond to even brightest light stimuli [11, 95]. Yet, the photoreceptors in *rd3* mice degenerate much faster than in the RetGC-deficient mice [95]. Importantly, the different degeneration patterns cannot be attributed to the differences in strain backgrounds, because both *rd3* and RetGC knockout mice in those studies were congenic with the C57B6 strain [95].

**Fig. 8:**
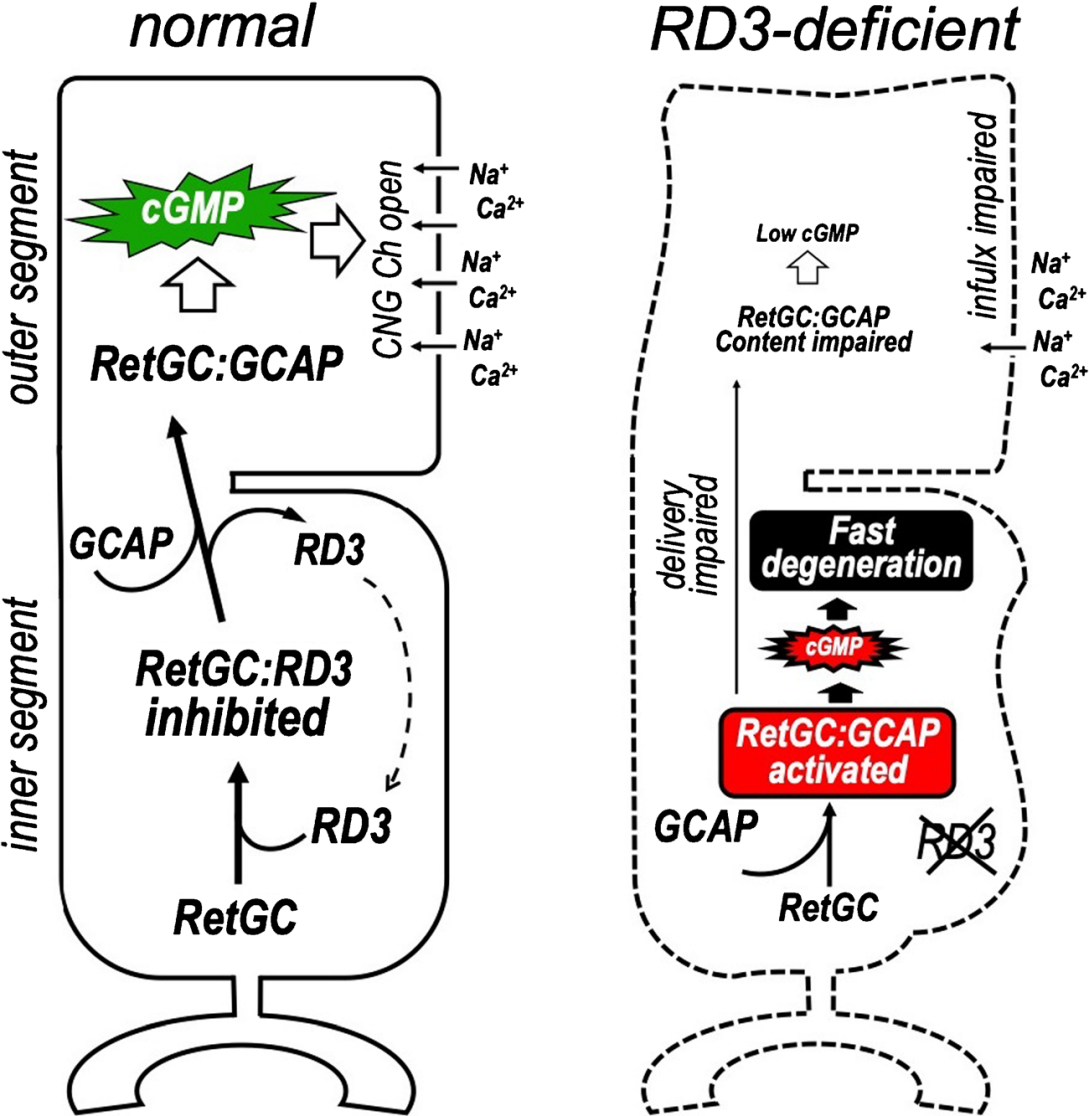
A proposed biological role of RD3 in photoreceptors [31, 74, 95]. RD3 stimulates the delivery of RetGC to the outer segment while blocking its activation by GCAPs in the inner segment. In RD3-deficient photoreceptors, the content of RetGC in the outer segment declines and the photoresponses become compromised due to insufficient production of cGMP; aberrant activation of the unprotected RetGC occurs in the inner segment and triggers degeneration of the photoreceptor cell

In addition to the support of the normal content of RetGC in photoreceptor outer segment, RD3 also has no less important second role in the physiology of photoreceptors, which is protecting them from degeneration. This is directly related to another main property of RD3, which is inhibition of RetGC activity [88]. RD3 strongly suppresses catalytic activity of RetGC1 and RetGC2 and also blocks their stimulation by Mg^2+^ GCAPs [88, 95]. RD3 demonstrates nearly 1000-fold higher apparent affinity for RetGC than GCAPs in vitro, such that RD3 at nanomolar concentrations outcompetes GCAP1 and GCAP2 present at the micromolar concentrations required to effectively stimulate RetGC [88, 95]. Importantly, the RD3-dependent inhibition does not change the Ca^2+^ sensitivity of the RetGC:GCAP complex, but rather inactivates the complex entirely [88]. Recent in vivo studies in transgenic mice strongly indicate that the inhibitory function of RD3 plays the principal role in protecting photoreceptors from *rd3* degeneration. After deletion of GCAP1 and GCAP2, the cyclase activity in *rd3* retinas declines even further, because now the cyclase is no longer activated by GCAPs at low Ca^2+^. Consequently, their ERG photoresponses are further suppressed as well [31]. Yet, quite surprisingly, the vast majority of GCAP-deficient *rd3* photoreceptors remain preserved at the ages when photoreceptors in *rd3* mice expressing GCAPs have already died out [31, 99]. These results argue that photoreceptors need RD3 to block RetGC activation by GCAPs that may occur in the wrong place and/or at the wrong time. The intracellular localization of RD3—predominantly in the inner segment [31, 99]—further supports the hypothesis that the most likely function of RD3 is to suppresses aberrant activation of RetGC in the inner segment, before the cyclase reaches its proper destination in the outer segment and becomes a part of the Ca^2+^ feedback regulating phototransduction in the outer segment (Fig. 8).

**Fig. 9:**
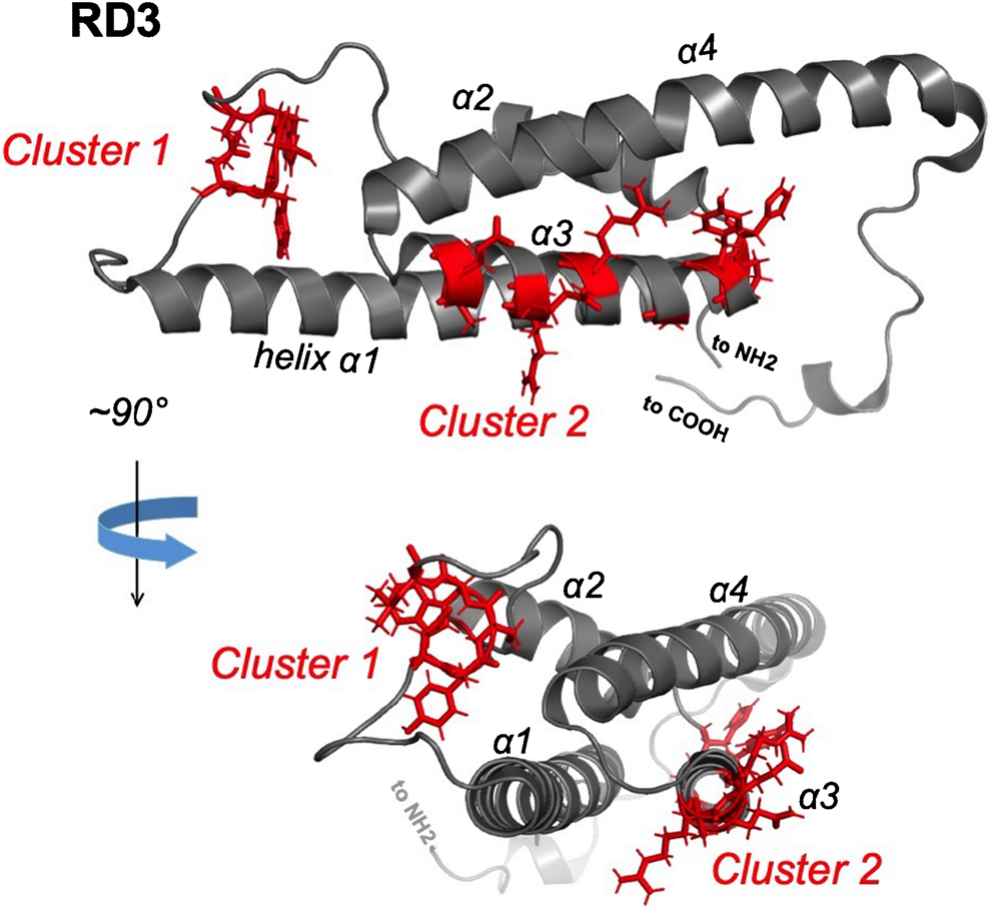
Ribbon diagram of the three-dimensional RD3 structure [97]. The two clusters of surface-exposed residues critical for the high-affinity inhibitory binding of RD3 to RetGC [85] are shown as sticks highlighted in red

Three-dimensional NMR structure of RD3 [97] shows a rather unique fold of this protein, with an elongated bundle of four alpha-helices as its central core adjacent to non-structured regions at the N- and C-termini (Fig. 9). Site-directed mutagenesis probing the entire surface of the molecule by point mutations [85, 95] has identified two main clusters of the surface-exposed side chains that enable the high-affinity binding of RD3 to RetGC: one in the loop connecting helices α1 and α2, and the other on the surface of the helix α3 (Fig. 9) [85]. It is much less clear where the interface for binding RD3 is located on RetGC. The removal of a short C-terminal fragment from RetGC1 disrupts binding of RD3 [10, 98] but not of GCAPs [93, 94, 98]. Conversely, substitutions in the cyclase dimerization domain can block the binding of GCAPs but not of RD3 [93]. On the other hand, some point mutations in the RetGC1 kinase homology domain disrupt binding of both RD3 and GCAP [93, 98]. Therefore, it is possible that while competing with each other for the cyclase, RD3 and GCAP use different binding interfaces on RetGC that either partially overlap within the quaternary structure of the complex or the two different binding sites can affect each other indirectly by rearranging the quaternary structure of the cyclase. This and many other outstanding questions about the molecular dynamics of RetGC in its complexes with RD3 and GCAP remain to be addressed in future studies.

## Does RD3 contribute to the photoreceptor degenerations caused by mutations in RetGC1 and GCAP1?

Recent studies also indicate that a CORD6-related substitution in the cyclase dimerization domain, Arg838Ser, not only increases RetGC1 affinity for Mg^2+^ GCAP and reduces its sensitivity to deceleration by Ca^2+^, but also makes the Mg^2+^ GCAP1: RetGC1 complex more resistant to inhibition by RD3 [30]. The most probable reason why higher concentrations of RD3 are required in that case is the increased affinity of the mutant RetGC1 for GCAP, which makes it more difficult for RD3 to displace GCAP from the complex with the cyclase. Likewise, a substitution in GCAP1 that also causes a dominant retinopathy, Gly86Arg, not only shifts the Ca^2+^ sensitivity of RetGC deceleration to a higher Ca^2+^ range, but also makes RetGC better resist the inhibition by RD3, again, most likely because of the increased affinity of the Gly86Arg GCAP1 for RetGC1 [96]. If the main role of RD3 is protecting photoreceptors against an aberrant activation of RetGC by GCAPs in the inner segment, then in the cases with both Arg838Ser RetGC1 and Gly86Arg GCAP1, the protective function of RD3 can be weakened by the higher stability of the GCAP:RetGC complex. This could present another factor contributing to the severity of retinal degeneration. Further studies should evaluate this possibility.
