# Correction to: Regulation of retinal membrane guanylyl cyclase (RetGC) by negative calcium feedback and RD3 protein

**DOI:** 10.1007/s00424-021-02547-w

**Published:** 2021-03-05

**Authors:** Alexander M. Dizhoor, Igor V. Peshenko

**Affiliations:** Pennsylvania College of Optometry, Salus University, 8360 Old York Road, Elkins Park, PA 19027 USA

**Correction to: Pflügers Archiv - European Journal of Physiology (2021)**

10.1007/s00424-021-02523-4

The article Regulation of retinal membrane guanylyl cyclase (RetGC) by negative calcium feedback and RD3 protein, written by Alexander M. Dizhoor and Igor V. Peshenko, was originally published electronically on the publisher’s internet portal on 3 February 2021 without open access. With the author(s)’ decision to opt for Open Choice, the copyright of the article changed on 24 February 2021 to © The Author(s) 2021 and the article is forthwith distributed under a Creative Commons Attribution 4.0 International License, which permits use, sharing, adaptation, distribution and reproduction in any medium or format, as long as you give appropriate credit to the original author(s) and the source, provide a link to the Creative Commons licence, and indicate if changes were made. The images or other third party material in this article are included in the article’s Creative Commons licence, unless indicated otherwise in a credit line to the material. If material is not included in the article’s Creative Commons licence and your intended use is not permitted by statutory regulation or exceeds the permitted use, you will need to obtain permission directly from the copyright holder. To view a copy of this licence, visit http://creativecommons.org/licenses/by/4.0.

The original article has been corrected.

**Open Access** This article is licensed under a Creative Commons Attribution 4.0 International License, which permits use, sharing, adaptation, distribution and reproduction in any medium or format, as long as you give appropriate credit to the original author(s) and the source, provide a link to the Creative Commons licence, and indicate if changes were made. The images or other third party material in this article are included in the article’s Creative Commons licence, unless indicated otherwise in a credit line to the material. If material is not included in the article’s Creative Commons licence and your intended use is not permitted by statutory regulation or exceeds the permitted use, you will need to obtain permission directly from the copyright holder. To view a copy of this licence, visit http://creativecommons.org/licenses/by/4.0/.

